# Alpha Lipoic Acid Attenuates Radiation-Induced Thyroid Injury in Rats

**DOI:** 10.1371/journal.pone.0112253

**Published:** 2014-11-17

**Authors:** Jung Hwa Jung, Jaehoon Jung, Soo Kyoung Kim, Seung Hoon Woo, Ki Mun Kang, Bae-Kwon Jeong, Myeong Hee Jung, Jin Hyun Kim, Jong Ryeal Hahm

**Affiliations:** 1 Department of Internal Medicine, Gyeongsang National University School of Medicine, Jinju, Gyeongnam, Republic of Korea; 2 Department of Otolaryngology, Gyeongsang National University School of Medicine, Jinju, Gyeongnam, Republic of Korea; 3 Department of Radiation Oncology, Gyeongsang National University School of Medicine, Jinju, Gyeongnam, Republic of Korea; 4 Biomedical Research Institute, Gyeongsang National University Hospital, Jinju, Gyeongnam, Republic of Korea; 5 Institute of Health Science, Gyeongsang National University School of Medicine, Jinju, Gyeongnam, Republic of Korea; IIT Research Institute, United States of America

## Abstract

Exposure of the thyroid to radiation during radiotherapy of the head and neck is often unavoidable. The present study aimed to investigate the protective effect of α-lipoic acid (ALA) on radiation-induced thyroid injury in rats. Rats were randomly assigned to four groups: healthy controls (CTL), irradiated (RT), received ALA before irradiation (ALA + RT), and received ALA only (ALA, 100 mg/kg, i.p.). ALA was treated at 24 h and 30 minutes prior to irradiation. The neck area including the thyroid gland was evenly irradiated with 2 Gy per minute (total dose of 18 Gy) using a photon 6-MV linear accelerator. Greater numbers of abnormal and unusually small follicles in the irradiated thyroid tissues were observed compared to the controls and the ALA group on days 4 and 7 after irradiation. However, all pathologies were decreased by ALA pretreatment. The quantity of small follicles in the irradiated rats was greater on day 7 than day 4 after irradiation. However, in the ALA-treated irradiated rats, the numbers of small and medium follicles were significantly decreased to a similar degree as in the control and ALA-only groups. The PAS-positive density of the colloid in RT group was decreased significantly compared with all other groups and reversed by ALA pretreatment. The high activity index in the irradiated rats was lowered by ALA treatment. TGF-ß1 immunoreactivity was enhanced in irradiated rats and was more severe on the day 7 after radiation exposure than on day 4. Expression of TGF-ß1 was reduced in the thyroid that had undergone ALA pretreatment. Levels of serum pro-inflammatory cytokines (TNF-α, IL-1ß and IL-6) did not differ significantly between the all groups. This study provides that pretreatment with ALA decreased the severity of radiation-induced thyroid injury by reducing inflammation and fibrotic infiltration and lowering the activity index. Thus, ALA could be used to ameliorate radiation-induced thyroid injury.

## Introduction

Radiation therapy is used widely in the management of head and neck tumors, lymphomas and malignancies of the central nervous system. Although the thyroid gland is not usually the radiation target, exposure of the thyroid to radiation during radiotherapy of the head and neck is often unavoidable. Hypothyroidism after radiotherapy for head and neck cancer was first reported in the 1960s [1]. Since then, many publications have described radiotherapy-induced thyroid disorders such as hypothyroidism, thyroiditis, Graves' disease, adenoma, and carcinoma [2]–[4].

Many methods of preventing and ameliorating radiation damage to normal tissues have been attempted, including using only the minimum dosage of radiation, hyper-fractionation, minimizing the radiation field, shielding, and using a radioprotective agent [5]–[9].

Risk factors for thyroid dysfunction include young age, high radiation dose, and history of thyroidectomy [4], [10], [11]. Additionally, the radiation-susceptibility of the thyroid was increased with an elevated level of thyroid-stimulating hormone (TSH) in rats [12]. Based on this observation, studies for TSH suppression by L-thyroxine (T4) have been performed, with controversial results. One study found that although hypothyroidism-free survival rates were higher in an adequately TSH-suppressed group compared to an inadequately TSH-suppressed group (70% vs. 20%, p = 0.02), the thyroid parenchyma was not influenced by TSH suppression [13]. Another study demonstrated that administration of T4 during radiation exposure did not prevent hypothyroidism, although the follow-up duration was insufficient to explain the preventive effect of thyroid tumors [14]. van Santen et al. [15] demonstrated T4 didn't protect against radiation-induced thyroid damage in rat model. No known drug has been shown to prevent radiation-induced thyroid damage. Thus, further studies should be required for finding a new drug.

Irradiation produces a cascade of free radicals, and antioxidant compounds have long been used to protect against the resulting radiation toxicity. Glutathione (GSH) elevating agents such as N-acetylcysteine (NAC) and α-lipoic acid (ALA), both of which are non-toxic (within certain concentration ranges) to humans, can protect normal tissues against radiation damage [16]–[18]. ALA is a strong antioxidant with high reactivity to free radicals that facilitates regeneration of vitamin C and E and elevates tissue levels of GSH [19]. ALA has been demonstrated to be effective in preventing pathological processes in which reactive oxygen species (ROS) have been implicated, such as ischemia-reperfusion injury [20], diabetes [21], hypertension, radiation injury [22] and HIV activation [23]. ALA has been shown to protect against radiation injury in mouse hematopoietic tissues and has increased the LD50 from 8.6 to 10.9 Gy with a dose-reduction factor (DRF) of 1.26 [24]. Furthermore, ALA treatment for 28 days lowered lipid peroxidation in children chronically exposed to low doses of radiation by the Chernobyl nuclear accident [17].

The present study aimed to investigate the protective effect of ALA on radiation-induced thyroid injury in rats.

## Materials and Methods

### Ethics Statement

The experiments were approved (GLA-120120-R0002) by the Gyeongsang National University Institution Animal Care & Use Committee.

### Animals and radiation exposure

This study was conducted using male Sprague–Dawley (SD) rats (230–250 g; Koatech Inc., Peongtaek, Korea). Animals were housed in temperature-controlled conditions under a light/dark photocycle with food and water supplied ad libitum. The experiments were approved (GLA-120120-R0002) by the Gyeongsang National University Institution Animal Care & Use Committee. Rats were randomly assigned to four groups: healthy controls (CTL, n = 12), irradiated (RT, n = 16), received ALA before irradiation (ALA + RT, n = 16), and received ALA only (ALA, n = 12). Rats were treated with either ALA (100 mg/kg, i.p., Bukwang Pharmaceutical Company (Seoul, South Korea) or saline (equal volume, i.p.) at 24 h and 30 minutes prior to irradiation. The ALA dose of 100 mg/kg was based on our preliminary trials and previous studies [27]. Rats were anesthetized to immobilize them prior to radiation exposure. The neck area including the thyroid gland was evenly irradiated with 2 Gy per minute (total dose of 18 Gy) using a photon 6-MV linear accelerator (21EX, Varian, Palo Alto, CA, USA). A 3-cm block of Lucite was positioned above the head and neck to provide adequate buildup and facilitate even radiation distribution. Each rat is exposed to a single dose of radiation and this method was modified from previous studies.

### Clinical examination

The radiation sites of all rats were evaluated for body mass, fur condition (decreased gloss, fading, upright hairs) and skin changes (alopecia, inflammation, wounds).

### Tissue preparations

Rats were humanely euthanized at 4, 7, 14 and 28 days after head and neck radiation. Blood samples were obtained via direct ventricular puncture to measure serum TNF-α, IL-1ß and IL-6 levels. The thyroid gland, salivary glands, and oral mucosa were then removed. Both thyroid lobes were removed en bloc together with the adjacent trachea to prevent surgical damage of the tissue. For western blot analysis, the right lobe of the thyroid was excised and stored at 80°C until use. The left lobe of the thyroid was removed for histological examination.

### Histopathological evaluation

Tissue samples were fixed in 4% paraformaldehyde in 0.1 M phosphate-buffered saline (PBS), embedded in paraffin, and cut into 5-µm sections. To assess the degree of inflammation and fibrosis after irradiation in each group, sections were stained with hematoxylin and eosin (H&E), periodic acid-Schiff (PAS; for glycoproteins) and Masson's trichrome, respectively. All tissue sections were examined microscopically for histopathological changes by an experienced histologist blinded to the study protocol. To score the thyroid gland activity, the following parameters were measured: follicular size, colloid density and the height of the follicular epithelium, which were scored from 1 to 5 as follows [25]: 1) Follicular size was scored inversely; thus, the smaller the follicle, the higher the score; 2) colloid density was scored inversely; thus, the lower the density of the colloid, the higher the score; 3) cell height was scored directly; thus, the higher the follicular epithelial cells, the higher the score. The activity index was calculated by adding all scores from the follicular size, colloid density, and cell height. A high index indicated active cell metabolism and a high protein turnover. A low index represented resting thyroid tissue with a large amount of thyroglobulin accumulated in the follicles. Additionally, the presence of follicular irregularity, hyperplasia, adenoma, cysts, carcinoma, and thyroiditis was evaluated.

### Immunohistochemical analysis

After deparaffinization, sections were sequentially treated with 1% H_2_O_2_ for 10 min and rinsed thoroughly with PBS. Sections were blocked with 2% normal goat serum in PBS at room temperature for 60 min to minimize nonspecific IgG binding, followed by incubation with rabbit anti-TGF-ß1 (diluted 1∶50; Santa Cruz Biotechnology, Santa Cruz, CA, USA), rabbit anti–8-OHdG (diluted 1∶500; abcam, Cambridge, MA, USA), and – mouse anti–MDA (diluted 1∶500; abcam, Cambridge, MA, USA). The samples were washed with PBS, incubated for 60 min at room temperature with biotin-conjugated secondary IgG (diluted 1∶200; Vector Laboratories, Burlingame, CA, USA), diluted in 2% normal blocking serum, and then incubated for 60 min at room temperature with avidin-biotin-peroxidase complex (ABC Elite kit, Vector Laboratories). The samples were then washed with PBS and incubated for 3 min with diaminobenzidine tetrahydrochloride containing 0.05% hydrogen peroxidase to develop the color. Samples were then stained with Mayer's hematoxylin stain. The sections were visualized under light microscopy and digital images were captured and analyzed.

### Measurement of serum TNF-α, IL-1ß and IL-6 levels

Plasma levels of TNF-α, IL-1ß and IL-6 were quantified using specific ELISA kits according to the manufacturer's instructions (Biosource International, Nivelles, Belgium).

### Statistical analysis

All statistical analyses were performed using the Statistical Package for the Social Sciences (SPSS, version 12.0, SPSS Inc., Chicago, IL, USA). Results are presented as means ± standard error of the mean. Differences between groups were assessed using one-way analysis of variance, followed by Student–Newman-Keul tests for pairwise multiple comparisons. A probability (P) value of <0.05 was considered to indicate a significant difference.

## Results

### Clinical findings during follow-up

The mean weight of rats in irradiated groups (RT and ALA + RT) was decreased after irradiation and was lowest at 11 days after irradiation (mean, 222.3±18.8 g). Intake of food and water was also diminished after irradiation. There was no difference in body mass between the RT only and ALA + RT groups. All rats had recovered at 12 days post-irradiation, after which body mass and food intake increased gradually (data not shown). Sustained increase in body weight was also seen in the non-irradiated control rats (Control and ALA only). From third day after irradiation, irradiated animals (RT and ALA + RT) showed hair loss at the radiation site and inflamed crusted periorbital skin. One rat in the RT group had severe body-mass loss (185.0 g) at 11 days post-radiation and gained weight gradually thereafter.

### Histological effects of ALA in thyroid tissues after radiation

**Figure 1:**
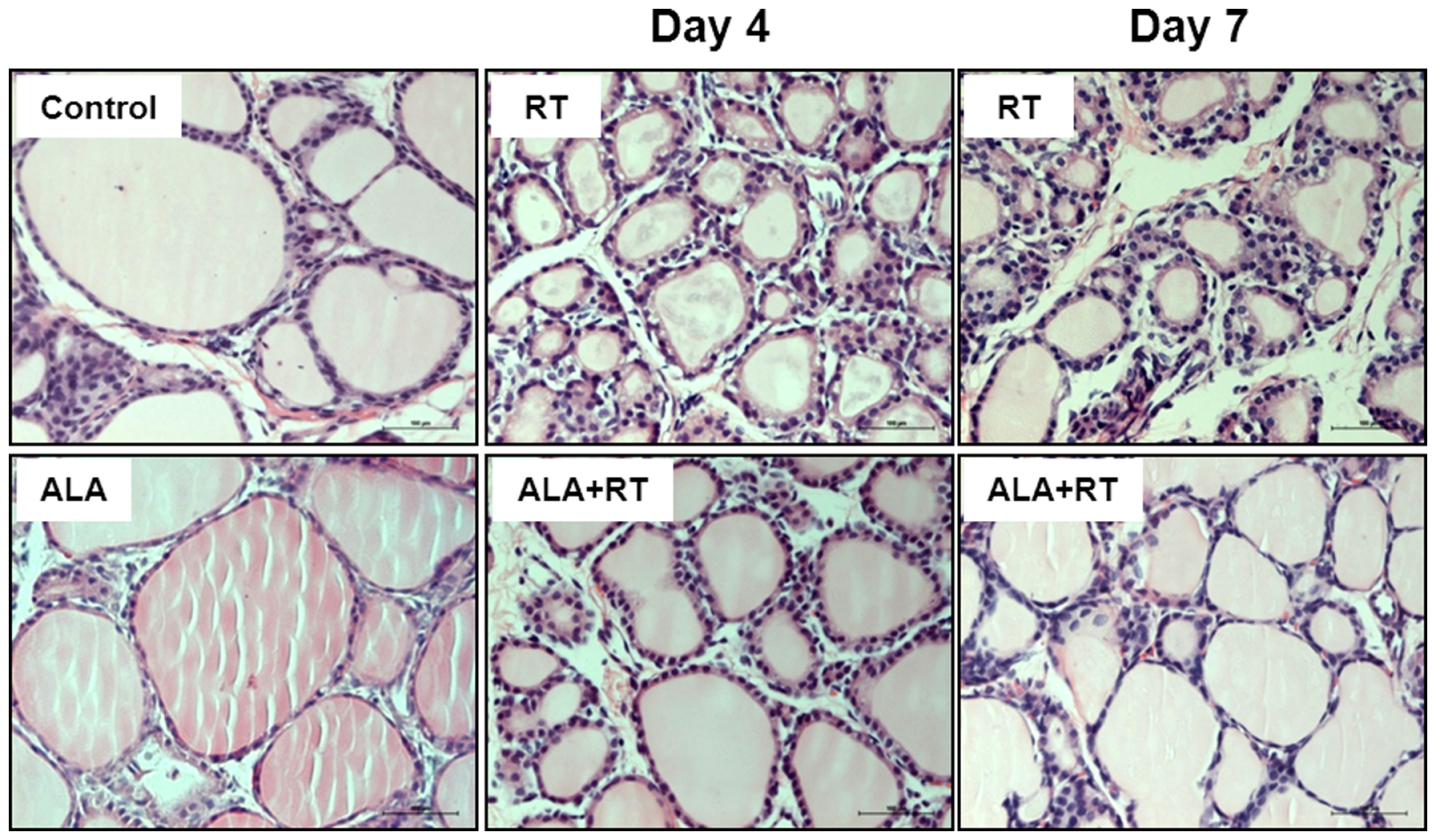
Representative photomicrographs of thyroid histology in the control, ALA, RT and ALA + RT groups after radiation. Hematoxylin-eosin staining was performed on cross sections of the thyroid glands. Irregular-sized, smaller follicles than in the control group are noted in addition to inflammatory cell infiltration in the interfollicular area in the irradiated thyroid. Follicles of the ALA + RT group are more normal in size than those of the irradiated rats. CTL, control. RT, irradiated. ALA, ALA only. ALA + RT, received ALA before irradiation. Scale bar, 100 µm.

Normal thyroid morphology was observed in the control and ALA-treated (ALA) groups. Follicles were lined by normal cuboidal epithelium in the control and ALA groups (Fig. 1). Greater numbers of abnormal and unusually small follicles were observed in the thyroid tissues of rats subjected to radiation (RT) compared to the controls and the ALA group. Follicles in the irradiated thyroid tissues were surrounded by cuboidal or columnar epithelium on days 4 and 7 and inflammatory cells were observed in the interfollicular areas in these tissue samples (Fig. 1). However, all pathologies were decreased by ALA pretreatment (ALA + RT) (Fig. 1 RT vs. ALA + RT).

### Effect of ALA on fibrosis in the irradiated thyroid

**Figure 2:**
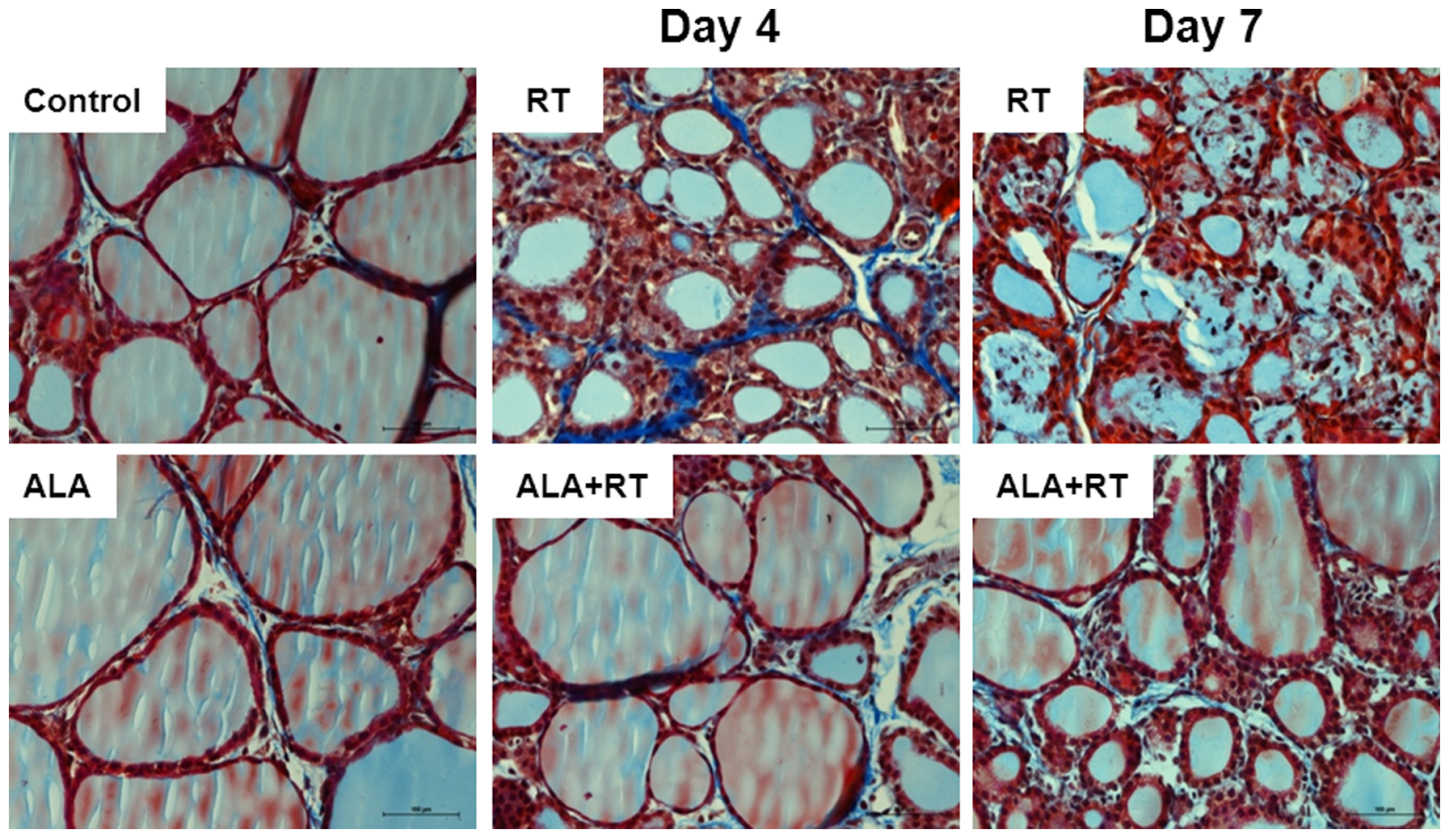
Representative photomicrographs of thyroid histology in the control, ALA, RT and ALA + RT groups after radiation. Masson's trichrome staining was performed on cross sections of the thyroid glands. There was severe fibrosis in the interfollicular space and interfollicular edema was present in the irradiated rat thyroid on days 4 and 7 post-radiation (RT). Irradiation-induced histological changes were attenuated by ALA (ALA + RT). CTL, control. RT, irradiated. ALA, ALA only. ALA + RT, received ALA before irradiation. Scale bar, 100 µm.

To determine whether ALA effectively inhibits radiation-induced collagen accumulation and fibrosis of the thyroid gland, we performed Masson's trichrome staining. Follicles in the control and ALA-only rats showed little Masson's trichrome staining but the thyroid of irradiated rats contained more connective tissue in the interfollicular spaces (Fig. 2). On day 4 after radiation, interfollicular edema and fibrosis were increased in the irradiated rats; the follicular structure was severely disrupted by day 7. However, the ALA + RT group showed little interfollicular edema or fibrosis.

### Effect of ALA on inflammatory cell infiltration into the irradiated thyroid

On days 4 and 7 post-irradiation, structurally damaged follicles and infiltration of inflammatory cells (including neutrophils and lymphocytes) into the interfollicular space were observed. Leukocyte infiltration was also observed in the connective tissue surrounding the follicles and in the follicular lumen in a few rats (Figs. 1 and 2). In the ALA-treated irradiated rats, leukocytes were rarely present in the interfollicular space.

### Effect of ALA on activity index in irradiated thyroid

#### Follicular size

**Figure 3:**
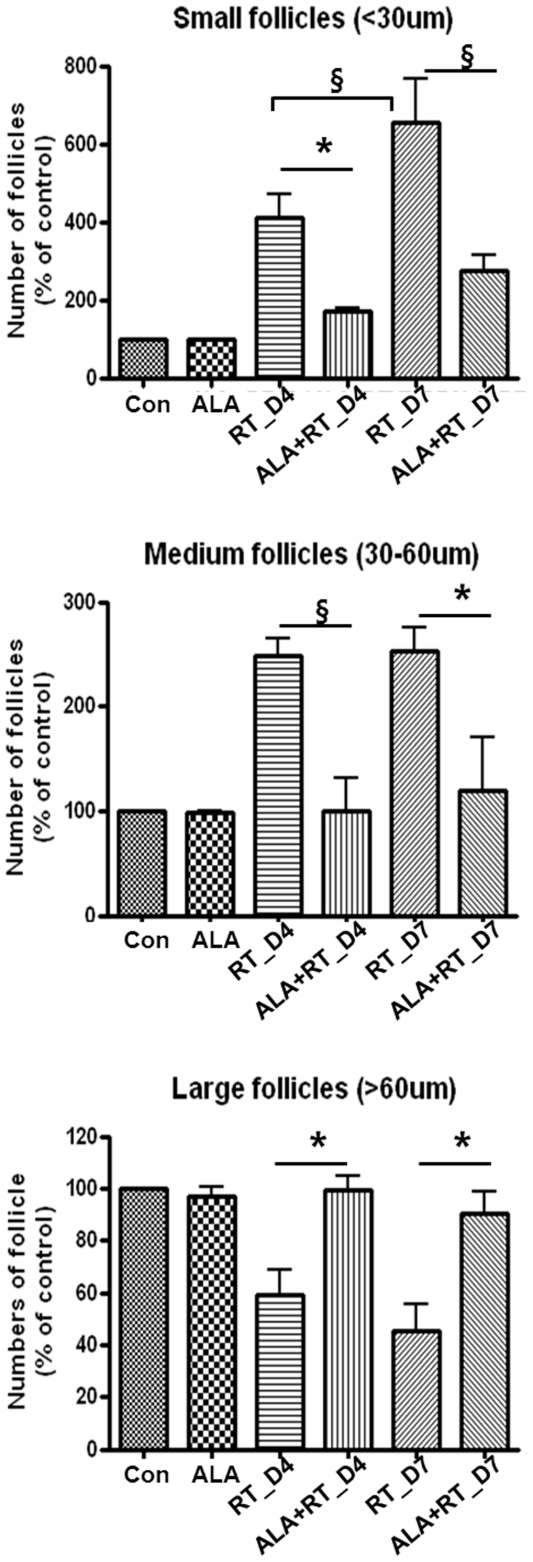
The percentage of three size-classes of the follicles. Follicles were divided into three categories based on their size: small (18–30 µm in diameter), medium (31–60 µm), and large (>60 µm). In the irradiated rats (RT), there occurred a statistically significant increase in the quantity of small and medium follicles compared to in the control and ALA-treated irradiated rats (ALA + RT). Small follicles were more common in the irradiated rats (RT), and greater numbers were observed on day 7 post-radiation compared to day 4. Small follicles in ALA-treated irradiated rats (ALA + RT) were less frequent than in irradiated rats (RT). (CTL, control; ALA, ALA only; RT-D4, Irradiated rats on day 4; RT-D7, Irradiated rats on day 7; ALA + RT-D4, ALA-irradiated rats on day 4; ALA + RT-D7, ALA-irradiated rats on day 7) (*P<0.05, §P<0.01).

The thyroid follicles were divided into three classes according to size: small (18–30 µm in diameter), medium (31–60 µm) and large (>60 µm) [26]. The quantities of small and medium follicles were significantly increased while the quantity of large follicles in the irradiated rats (RT) was decreased significantly compared to the control group (P<0.05 for both). Moreover, the quantity of small follicles in the irradiated rats was greater on day 7 than day 4 after irradiation (P<0.05, RT_D4 vs. RT_D7). However, in the ALA-treated irradiated rats (ALA + RT), the numbers of small and medium follicles were significantly decreased to a similar degree as in the control and ALA-only groups (Fig. 3). The percentage of follicles of all classes did not differ significantly between the control and ALA-treated irradiated rats (ALA + RT).

#### Colloid density

**Figure 4:**
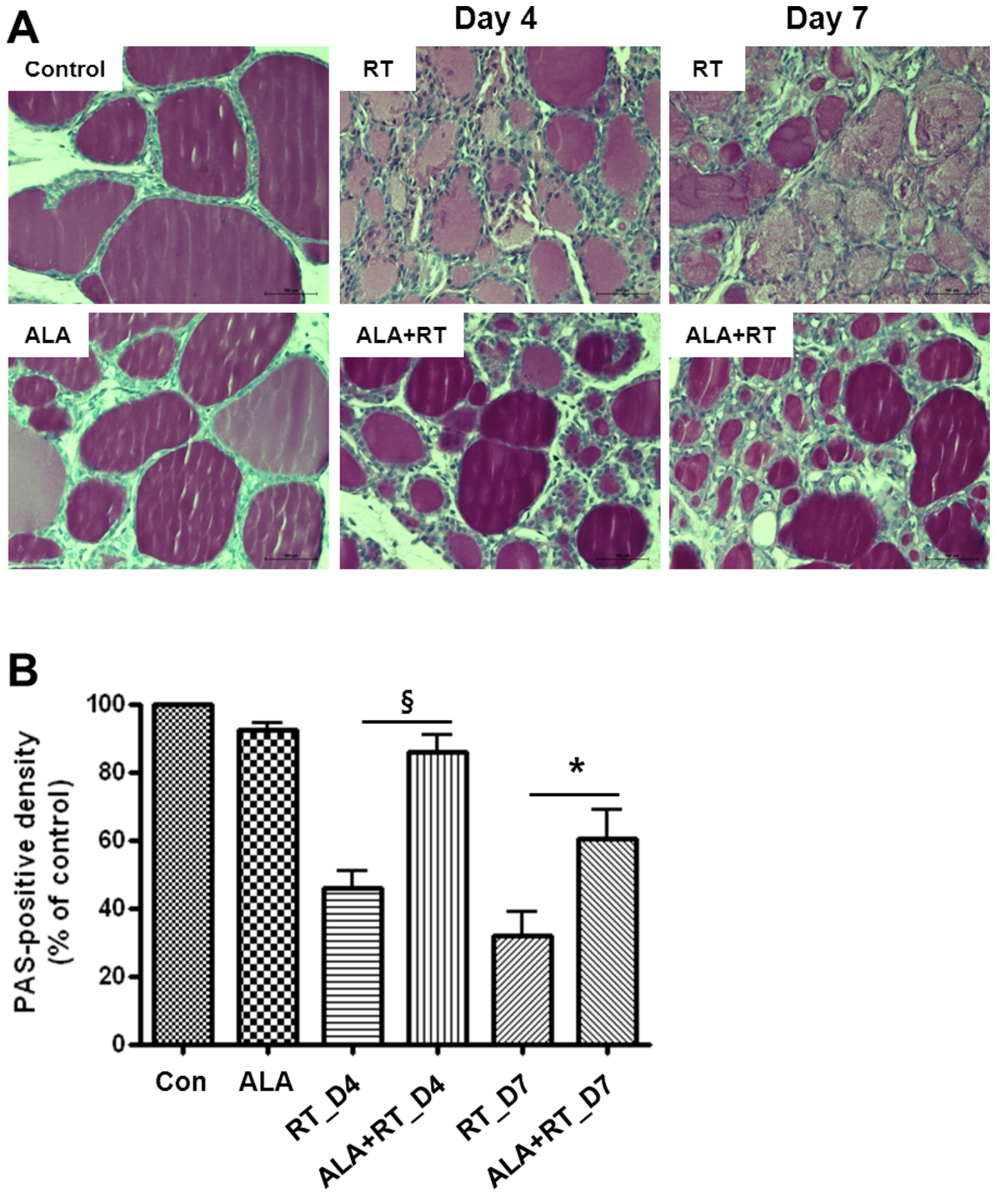
A. Representative photomicrographs of thyroid histology in the control, ALA, RT and ALA + RT groups after radiation. PAS staining was performed on cross sections of thyroid gland tissue. Follicles in the control rats were of regular size and had intensely stained colloid, indicating a low activity index. Follicles in the ALA-treated rats (ALA) without irradiation show similar degrees of colloid to the control group. The follicles were filled with less intensely stained colloid and were smaller than those in the control group, indicating a higher activity index. The follicles were filled with dense colloid similar to the controls in the ALA-treated irradiated rats. CTL, control. RT, irradiated. ALA, ALA only. The ALA + RT group received ALA before irradiation. Scale bar, 100 µm. **B**. PAS-positive colloid density. The PAS-positive density of the colloid in irradiated rats (RT) was decreased significantly compared with in all other groups. ALA-treated rats (ALA) showed a similar density of PAS-positive colloid to control rats (*P<0.05, §P<0.01).

These normal or regular follicles are filled with colloid, which usually stains pink with PAS. As shown in Fig. 4A, follicles in the control and ALA-only rats were of regular size and had intensely stained colloid. The follicles from irradiated rats were filled with less intensely stained colloid compared with the control and ALA-only rats. In the ALA-treated irradiated rats (ALA + RT), the follicles were filled with dense colloid similar to the controls, but some irregular and small-sized follicles remained. Figure 4B shows the PAS-positive density of the colloid in each group. ALA-treated rats (ALA) had a density of PAS-positive colloid similar to the control rats. The PAS-positive density of the colloid in irradiated rats (RT) was decreased significantly compared with all other groups (control, ALA, and ALA + RT). The decreased PAS-positive density of irradiated rats was reversed by ALA treatment (ALA + RT). However, the PAS-positive density was not significantly changed between days 4 and 7 in the irradiated rats (RT) or ALA-treated irradiated rats (ALA + RT).

#### Height of follicular epithelial cells

As shown results, sections from control and ALA alone by reflected with HE and PAS staining (Figs. 1, 2, and 4) showed well organized follicles such as each follicle is surround mainly simple cuboidal epithelium and is filled with a colloid. However, small and medium-sized follicles called radiated follicles showed a typical the active thyroid gland histology. Note that more typical vesicular nuclei, crowded nuclei, and the small, punched out “reabsorption lacunae” in the colloid next to the cells (Figs. 1, 2, and 4) and little colloid is visble and abundant stromal areas with fibrosis (Figs. 1, 2, and 4).

#### Activity index

Significant differences were found in the cell activity indices of the thyroid between the irradiated groups and the other groups (Figs. 1, 3, and 4). Irradiated rats (RT) had a higher activity index compared to the controls. However, the high activity index in the irradiated rats was lowered by ALA treatment (Fig. 4. RT vs. ALA + RT). No significant difference was found in the activity indices of the control and ALA groups (Fig. 4. Control vs. ALA).

### Effect of ALA on TGF-ß1 expression in the irradiated thyroid

**Figure 5:**
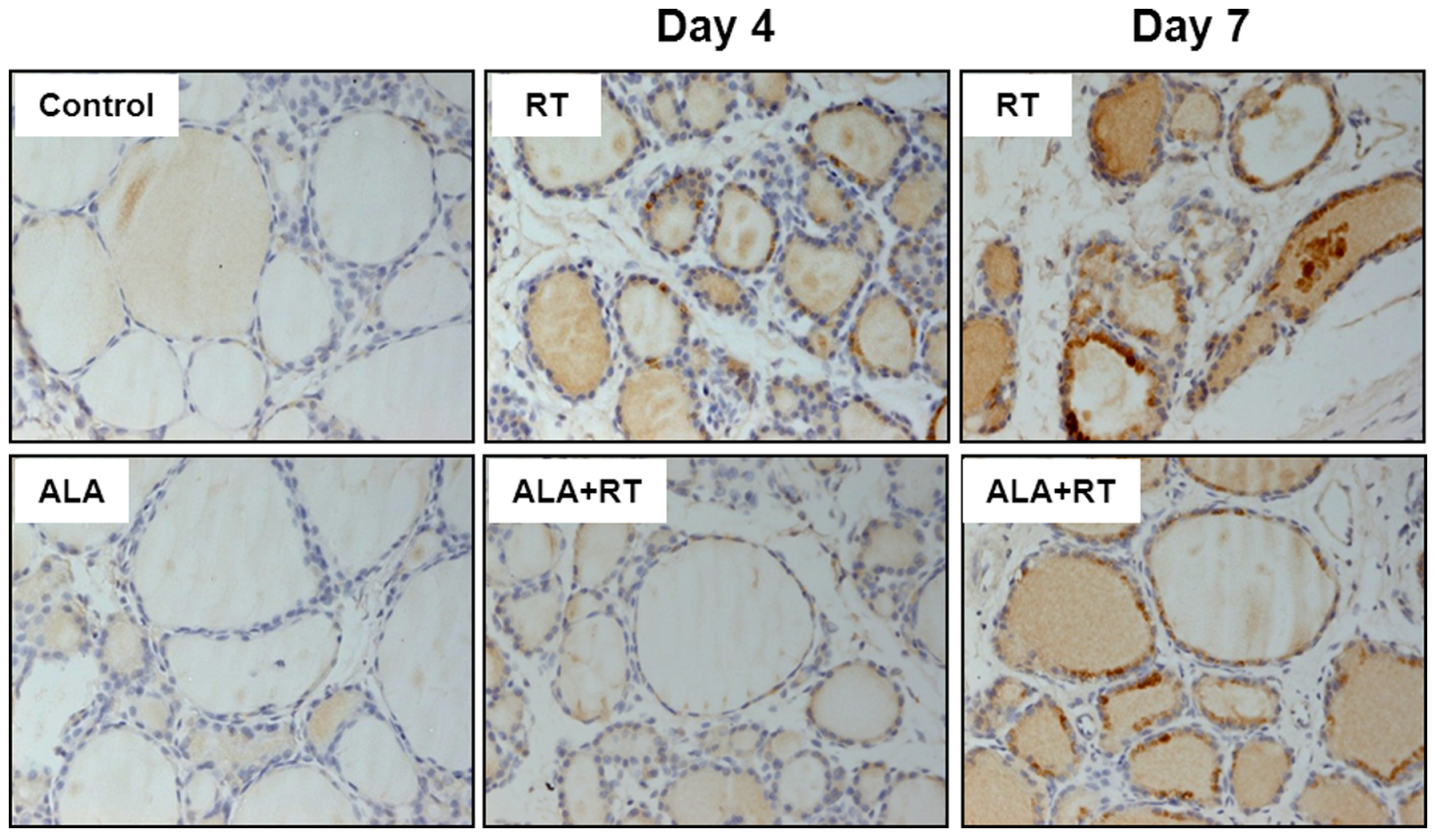
TGF-ß1 immunohistochemical staining of cross sections of rat thyroid gland tissue. The immunohistochemical localization of TGF-ß1 appears as dark-brown staining. TGF-ß1 expression was more intense in the follicular epithelium of the irradiated rats (RT) (thyrocytes strongly express TGF-ß1). The signals were stronger on day 7 than day 4. TGF-ß1 expression was less intense in the ALA group compared to the RT group. CTL, control. RT, irradiated. ALA, ALA only. ALA + RT, received ALA before irradiation. Scale bar, 100 µm.

Immunohistochemical staining of TGF-ß1 was used to evaluate whether ALA attenuates radiation-induced TGF-ß1 expression in the rat thyroid. Thyroid tissue sections from the control rats showed little expression of TGF-ß1 (Fig. 5. Control). TGF-ß1 immunoreactivity was enhanced in irradiated rats (RT) and was more severe on the day 7 after radiation exposure than on day 4. Expression of TGF-ß1 was reduced (ALA + RT) in the thyroid that had undergone ALA pretreatment. These results indicate that ALA could attenuate pro-inflammatory or fibrogenetic factor expression in thyroid tissue after irradiation.

We investigated the effect of ALA on serum pro-inflammatory cytokine levels in irradiated thyroids of rats. Levels of pro-inflammatory cytokines (TNF-α, IL-1ß and IL-6) did not differ significantly between the groups at any time point (data not shown); ALA treatment had no significant effect on the serum concentrations of these cytokines.

### Effect of ALA on oxidative stress in the irradiated thyroid

**Figure 6:**
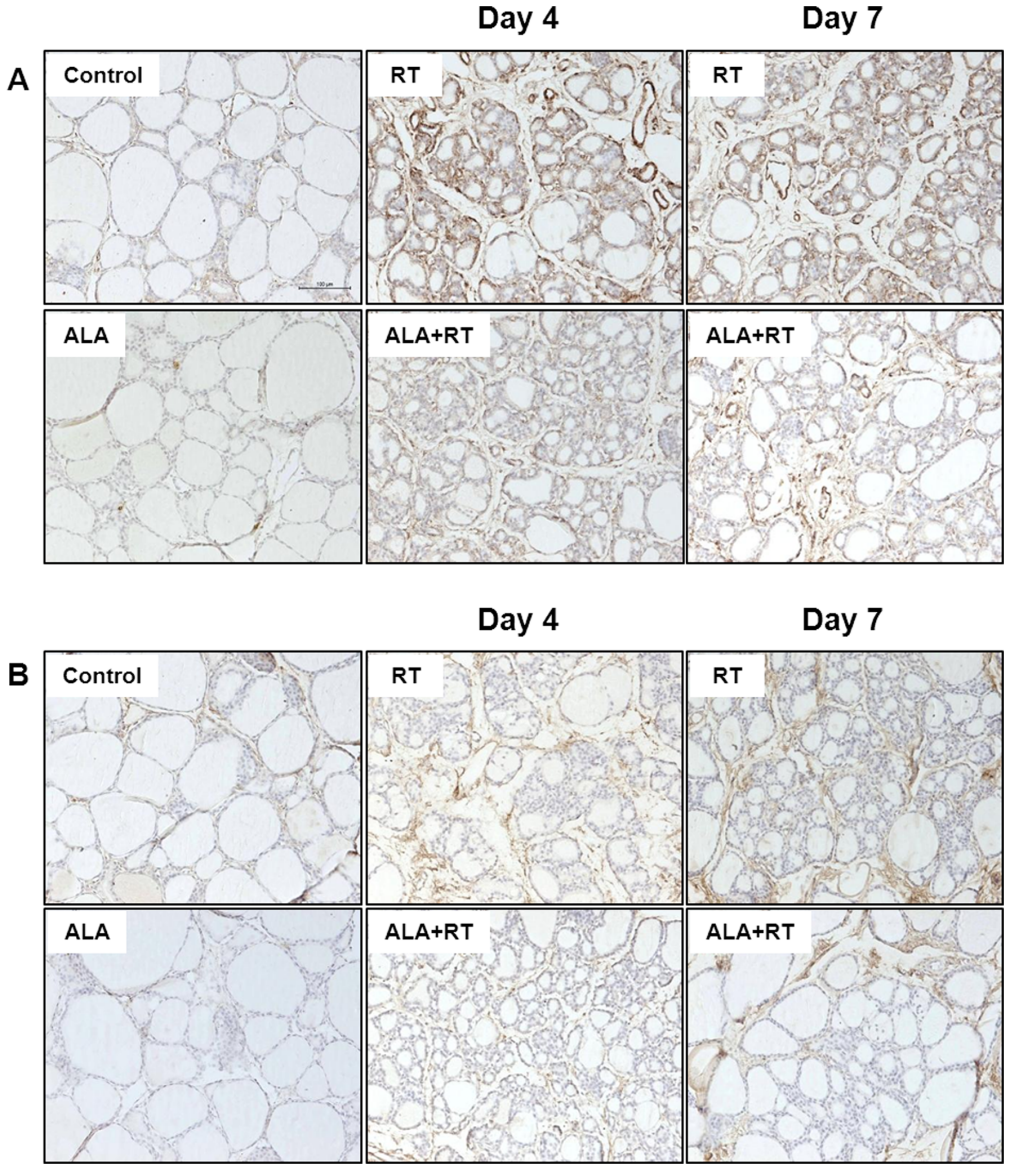
Immunohistochemical staining for 8-OHdG and MDA. 8-OHdG-positive signals were much dense at day 4 than day 7 after radiation (A). Expression of MDA was localized at the stromal area in radiation groups (B). Both signals were decreased in ALA-treated groups. Scale bar, 100 µm.

To investigate effect of ALA on radiation-induced oxidative stress, immunohistochemical staining of 8-OHdG and MDA was performed in all groups. 8-OHdG is a ROS-induced DNA damage marker and 8-OHdG-positive signals were much dense at day 4 than day 7 after radiation and were detected in the nuclei of irradiated follicular epithelial cells. The signals were decreased by ALA treatment at day 4 and 7 (Fig. 6A). Expression of MDA, lipid peroxidation marker, was localized at the stromal area in radiation groups and signals were decreased in ALA-treated groups (Fig. 6B).

## Discussion

The results of the current study demonstrate that irradiation of the head and neck induces inflammation and fibrosis of thyroid follicles with a high activity index. Pretreatment with the antioxidant ALA attenuates these pathologic changes, as indicated by the lowered activity index and TGF-ß1 expression.

The thyroid gland is a non-target organ during radiotherapy of head and neck cancers. Hypothyroidism is the most common sequelae and thyroid cancers can also develop after irradiation, especially in children [2]–[4]. The pathophysiologic characteristics of radiation-induced thyroid damage are related to inhibition of follicular epithelial function and subsequent progressive alteration of the endothelium. These sequelae are caused by cell degeneration and necrosis with follicular disruption in addition to vascular degeneration and thrombosis associated with acute and chronic inflammation, fibrous organization, and partial epithelial regeneration [28]. In our study, some follicles were collapsed and distorted with an irregular outline and had assumed a microfollicle configuration on days 4 and 7 after irradiation.

Ionizing radiation has been shown to enhance the production of ROS that can induce oxidative damage. Many antioxidant therapies have been used to overcome the potential harmful effect of free radicals and to reduce damage by oxidants. The representative radioprotective compounds are amifostine, NAC and ALA [16]–[18], . Particularly, ALA has been shown to effectively prevent pathology in various experimental models in which ROS are implicated, such as ischemia-reperfusion injury [20], diabetes [21], hypertension, radiation injury [22] and HIV activation [23]. In addition to ROS scavenging, ALA is associated with the recycling of other cellular antioxidants, including vitamins C and E, and GSH, which is referred to as the antioxidant of antioxidants [19]. Moreover, no toxicity has been reported at certain dose ranges [16], [30], [31]. This capability of ALA may alleviate systemic inflammation associated with oxidative stress. Hence, we used ALA pretreatment in the present study.

Previous studies have demonstrated that ALA attenuates x-irradiation-induced oxidative stress in mice by mitigating lipid peroxidation and protein oxidation [32] and that treatment with ALA protects tissues of mice from radiation toxicity [33]. ALA has also exhibited an anti-inflammatory effect on cotton pellet-induced chronic inflammation in rats [34]. One of the most important points of the current study is that the high activity index after irradiation was decreased significantly by ALA. The activity index provides specific information on thyrocytes [25]. Van Santen et al. suggested that the activity index is an objective indicator of the severity of radiation damage that should be used in studies on prevention of radiation damage in rats [25]. A high index indicates active cell metabolism and high protein turnover. A low index represents resting thyroid glands with considerable thyroglobulin accumulation in the follicles. Cells with the highest metabolic and mitotic activity after radiation exposure show the highest activity index and are at increased risk of developing neoplasms. In our study, PAS staining was slight due to low-density colloids and the activity index was high in irradiated rats (RT); however, ALA-treated irradiated rats (ALA + RT) showed dense colloid and a low activity index.

The fibrogenetic cytokine TGF-ß appears to be a common end-stage pathway in the development of tissue fibrosis in a variety of conditions [35]. In fact, the role of TGF-ß1 has been suggested based on its expression in the destructive granulomatous form of experimental autoimmune thyroiditis [36] and inhibition of TGF-ß1 by anti-TGF-ß1 antibodies or lisinopril reduced thyroid fibrosis in the same model [37]. TGF-ß can regulate epithelial cell growth and is over-expressed in thyroid follicular tumors and thyroid hyperplasia [38]–[40]. Thus, modulation of radiation-induced TGF-ß expression may attenuate fibrosis and cancer. The present study showed that TGF-ß expression and fibrosis after radiation were reduced by ALA pretreatment, indicating that ALA inhibits TGF-ß1 expression and could also prevent radiation-induced thyroid fibrosis and tumorigenesis. ALA also ameliorated radiation-induced oxidative stress reflected by 8-OHdG and MDA staining. 8-OHdG is a ROS-induced DNA damage marker and MDA is lipid peroxidation marker. Thus, these results suggest that ALA acts to prevent oxidative stress in the irradiated thyroid tissues.

The present study used the activity index to confirm that a radiation dose of 18 Gy is sufficient to induce active mitotic thyrocytes and that pretreatment with ALA can reduce mitotic activity, indicating that ALA may prevent malignant changes of the thyroid gland caused by long-term radiation. Saad et al. evaluated the Ki-67 proliferative index in the thyroid gland of various age groups, concluding that the decreased proliferative activity of thyroid cells with age could explain the higher incidence of radiation-related thyroid cancers in children than in adults [41]. Long-term follow up studies with younger animals are needed to evaluate the effectiveness of ALA in delayed malignancies after radiation. In future, the radio-protective effect of ALA against delayed radiation-induced thyroid damage should be evaluated.

In conclusion, the present study showed that pretreatment with ALA decreased the severity of radiation-induced thyroid injury by reducing inflammation, fibrotic infiltration, lowering the activity index, and oxidative stress. Thus, ALA treatment could ameliorate radiation-induced thyroid injury.
