# Correction: Alpha Lipoic Acid Attenuates Radiation-Induced Thyroid Injury in Rats

**DOI:** 10.1371/journal.pone.0131147

**Published:** 2015-06-17

**Authors:** Jung Hwa Jung, Jaehoon Jung, Soo Kyoung Kim, Seung Hoon Woo, Ki Mun Kang, Bae-Kwon Jeong, Myeong Hee Jung, Jin Hyun Kim, Jong Ryeal Hahm

In the Funding section, the grant number from funder Ministry of Education Science and Technology is listed incorrectly. The correct grant number is [2012R1A1A4A01015109].
